# A Dentalation

**Published:** 1881-02

**Authors:** J. Washington Clowes


### ARTICLE III.

### *A Dentalation as Recently Delivered before the Odontological Society of New York.*

BY J. WASHINGTON CLOWES.

My subject, this evening, is "MATERIALS FOR FILLING TEETH." There are several of these worthy of special mention: Gutta Percha, Gold, Os-artificial, Tin and

Amalgam; each very good in its appropriate place. In the olden time Gold was considered the only proper filling for the fangs of teeth, and, under this impression, many heroic attempts were made, some resulting in success, others in failure; but in the main, it was a worthy procedure, because the motive and the material were pure. In process of time, another substance was employed, which obtained consideration by reason of the great confidence reposed in the person who advised its use; but it has been proved a deception and a fraud, and its name is cotton! A fang thus filled will sooner or later come back to plague you, or your patient will be driven for relief into other hands. Cotton was retrogade, Gutta Percha is progress, and under the name of "Bevin's Stopping," is the most perfect fang filling in the world.

Gold! Ah, my friends, there is magic in the word. Gold! pure, unadulterated gold is accepted the world over as a standard filling; but it has its place. It is only second-rate for fangs. It often fails in large and shelly rearmost molars, and is not reliable in approximal cavities of difficult access. It is gold? It is precious gold! But we must not fall down and worship it!

Tin and Os-artificial have many commendable qualities, and while I acknowledge their merits, I cannot refrain from saying, they are at their best only, when covered by some material harder and more durable than themselves.

Amalgam! What shall I say? As I mention its name a throng of memories crowd upon my mind! I recollect its early associations, its imperfect material and preparation, and its mal-appliance. I recall the long list of evil names that have been given, and noxious qualities that have been ascribed to it; that it was once the cause of a fierce dissension in our professional ranks; that this dissension culminated in revolt; that the elemental and component parts of a great and learned association were dissevered, broken up and scattered; that the combatants, for and against Amalgam, fought exhaustively and long, and that (the great battles being over) a guerilla warfare still continues, on the part of the vanquished, from behind the cover of famous names, groundless prejudices, shameless calumny and popular alarm. Thirty-one years ago, Amalgam, as a filling for teeth, was brought to my notice. It was spoken of by Doctor Harris, of Baltimore, in most uncomplimentary language. By him the initial class of the first Dental College in the world, were taught that it was poisonous; they were admonished to abjure and refrain from it in their practice, and as they were taught so went they forth, to their various fields of labor, denouncing its users and condemning its use. In my office, during the first years of professional practice, poison, salivation, galvanic action, charlatan, quack, humbug, impostor, were words of frequent utterance, and the mere sight of an amalgam plug, in a patient's mouth, would excite my indignation, and arouse within me the spirit of antagonism and warning. It was on an occasion like this, at the close of a wordy crusade against the forbidden filling (my last javelin thrown and ammunition exhausted,) that my patient thus replied: "I have heard attentively all that you have said, and am disposed to leave the matter in your hands, but first desire to give you the history of this particular filling, and then will submit the case entirely to your judgment. Three years ago I called upon Dr. D——— in reference to the tooth that holds this plug, and asked his professional advice. After a long examination it was decided to be past saving, the nerve bare, the tooth itself a mere shell, and extraction the only proper and rational thing be done. I said: 'Oh, doctor, how can I part with my tooth? What shall I do without it? Do, doctor, think of some way to save it!' He replied: 'There is no way; I can do nothing,' and proceeded with his lance to sever the gum; this being done, I made a last appeal—with bleeding mouth, I entreated him to do something—anything, but allow me to lose my tooth. 'My dear lady,' he said, at length; 'there is one thing I can do, if you wish it. I do not approve of the means to be employed; but it may possibly help to keep your tooth for awhile; I promise you no real benefit from its use. It is called Amalgam, and is everywhere spoken against, and no dentist who is careful of his reputation would willingly use it. Shall I employ it?' 'Anything, anything,' was my answer, 'rather than part with my tooth.' He proceeded to remove merely the loose particles of food and decay from the cavity, and after preparing his filling in the same imperfect manner, applied it with the pressure of his finger. Except for about one week, subsequent to the operation, during which it was sensitive to heat and cold, it has been to me for the last three years a comfortable and useful tooth. Is it *really* poisonous, doctor? Will it really salivate me? Will it affect my health injuriously? Must you remove the filling that has proved to me so good a friend?" There are crises in our every life. They come to us by benign appointment. They are golden opportunities presented by invisible hands. We may recognize or ignore them, we may accept or reject them. But our decision must be prompt; they do not tarry. We must seize, we must lay hold upon them, when they appear, or they pass us by forever!

When my patient had closed her narration I felt there was a *crisis upon me:* the hand of progress had rolled back the veil of prejudice and error, and presented to my mental view possibilities and opportunities eminent and glorious! The pathway of usefulness was before me. It was mine to pursue it, and I did. The Amalgam plug, that had been imperfectly prepared and inserted, was removed, but only to be replaced by one improved by care and precision; for I reasoned, "if good has been accomplished by the incomplete and unskillful, what may not be achieved by better means and methods?" Many years after its refilling, the tooth, from which I had removed the imperfect plug, was still doing faithful service in the mouth of her who had appreciated and deserved it.

I dwell upon this case with peculiar emphasis; because it was the opening door through which I passed to a higher plane and clearer view. For more than a quarter of a century I have stood upon this plane, and with all the energy I possessed, have openly and fearlessly proclaimed the excellence of Amalgam. From the first moment of my full conviction of its worth, I have not failed in its advocacy. Amalgam was always a saving plug, and, in the march of dental progress, there is none that gives fairer promise of ultimate perfection.

The assertion of Dr. C. C. Allen was well and truthfully made, when he declared: "If my life and fortune depended upon the saving of a tooth merely, without regard to its appearance, I would fill it with Amalgam."

If such was the testimony of one of the fathers, in reference to this filling, in its beginning, what may not be said of it now in its advanced and improved condition? As I first saw it in the mouth, it presented a dark and uncomely appearance, which was invariably imparted to the substance of the tooth. It was black, but it was honest and true. If its ugliness was a reason for rejection, who then shall be received? As a preference for the beautiful, as well as the good, is commendable and wise, I resolved to strive for their attainment. My first step in this direction was in the employment of pure materials. The original coin filings, having an admixture of copper, were replaced by a precipitate of virgin silver; giving the result of a whiter, but more fragile filling. The next evidence of progress was the addition of a certain proportion of pure tin-foil to the precipitate. This lent toughness to the mass which, when applied imparted no discoloration to the dental walls, but was still subject to blackness on its face. To me this was matter for deep regret, and in those days—when my patients would cry out against the "darkness visible"—I never failed to remind them that they must not "speak ill of the bridge that carried them safe over," and as to the color it was only superficial,—a mere stain, that an appropriate dentifrice would readily remove. But I longed earnestly for the "good time coming," when no uncanny look should appear upon the surface of my work. That time has come. To Drs. Hunter and Lawrence its advent is mainly due, and to the latter especial praise belongs; his Amalgam, when skillfully applied, is one of the best fillings for the teeth of which I have any knowledge. May he live long, and strive continually to improve it even to perfection. Thus imperfectly have I discussed the merits, development and progress of Amalgam.

Conceived in weakness, brought forth and nurtured by empyricism, traduced, maligned, denounced by professionals in high places, malused, abused and buffeted, it has come to be a power in the land. Out of weakness it has grown strong, and the very vices of its origin, are covered by the mantle of its virtues! Its most potent enemies have been *misuse* and *disuse.* By the one, apparently abundant evidence has been given for its condemnation ; by the other, under cover of regard for the public weal, the attempt has been, and is, constantly made to bring it into disrepute. What shall I say of him who has villainously forced a metallic substance upon the exposed dental pulp ? How can I sufficiently execrate the wretch who crowds any filling into the fetid internal of a dead and ulcerated tooth ? From acts like these come irritations, inflammations, swellings, suppurations, nervous prostrations, and a host of other ills, the cause of which is apparent to *disuse* only when Amalgam has been employed ; but to the intelligent and candid mind is proved, as well as appear, to be the result of pressure and obstruction from any foreign source. Thus, contact and association, with dying and deceased dental pulps, have covered Amalgam with especial obloquy, and, like poor, peaceful dog Tray, it has suffered accordingly. Disuse, in the past, assumed its most formidable proportions, its very culmination of antagonism, in the person and assertions of one of the elder dentists. By what peculiar means, by what subtle influence, he was enabled to catch, absorb and control the public mind, I leave unsaid ; but as an alarmist, I may assert, that seldom has one appeared among men more persistent and terrible; like him of old who sat at the feet of Gamaliel, he persecuted with zeal, and its results have indeed been wide spread and appalling. When the startling cry of "fire! fire!" is shouted in the midst of multitudes that seek for safety in the mad rush toward a single and narrow exit; the trampling of the strong upon the weak, the shriek of agony, the smothered groan, the crackling flames, the blinding smoke, are scenes and sounds that mark the passing tragedy. All these shall have an end: but the alarm which goes abroad—that enters many peaceful homes, and with a ceaseless torture, distracts the mind and generates distrust—this is a living woe, a limitless calamity! As by one man's *ipse dixit*, this evil influence was set in motion so by many others of like temperament and equal capacity, it has been nourished and sustained. No words can tell, no pen portray the amount of mischief which the Great Alarmist and his pliant disciples have ignorantly wrought.

As when, beneath the Banyan's outstretched arms,
And sheltering shade, the tribes of men abide,
(Harboring no thought except of confidence.
Feeling no impulse save of gratitude,)
Content and peaceful thus, day after day,
In happy sequence, glides, and all is well!
Anon a rumor comes, borne on the air,
A startling rumor! At first so startling,
As scarce to receive the slightest credence,
Yet makes no pause; but most assiduous,
Still comes and goes until, at length its voice,
Insinuous with subtle guile, absorbs
The conscious sense and tensely list'ning ear!
Then consternation, vast and terrible,
Assumes control, and holds high carnival!
The well beloved and sheltering Banyan arms,
The far o'er-reaching shade—the home content,
The social joys—unnoticed press, unheeded call,
And forth from their embrace and kindly cheer,
The frighted habitants bewildered run;
*For rumor infamous, and vile, and base,*
*With still evenomed words and slanderous tongue,*
*The good old tree, a deadly Upas calls,*
*And is believed.*

The combination of metals known as Amalgam, is singular in this: That of all the faults it is said to possess, of all the harm it is said to have done, it is as free and innocent as the child unborn. It never did and it never can poison, salivate, galvanize, or injure any one in the least degree. As innoxious and digestible as a grape seed to swallow; it is as incapable of evil as the hand of friendship or the kiss of affection. I am impelled to take up its defense at this time by a sense of duty which I owe to it and to every people, wherever in the wide world the voice of its calumniators has been heard, believed, and had power to alarm. I know of no worthier act than the performance of duty, no higher aspiration than the enunciation of truth, no attainment more eminent than professional excellence. By the sense of duty well performed, by the truth fearlessly proclaimed, by the excellent idealized and labored for, I beseech you, brethren, be not henceforth faithless but believing.

What shall I say? Amalgam! How wonderful and mysterious the union it represents! How beautiful the chemical affinities that bind it! Was there ever before such a metallic blending as this? Pliant to manipulate, facile to apply; to weakness lending strength, to frailty support. Plastic in form—now moulded by the touch—yielding to slight pressure, penetrating and filling the minute, as well as the vast; entering into and hermetically barring the interior of a tooth against the attacks of its enemies. As we contemplate, we admire; while admiring, new wonders rise! The soft and ductile becomes hard and unyielding; the impressible a fixed and unchanging mass; inexpansive and unshrinking, how perfectly it does its work! If it lacked either of these qualities it would fail in its purpose. It does not lack, it does not fail; but in all the essentials that make up a saving plug it eminently abounds. There are general providences all around us; there are special ones as well. In the far-gone ages the great and good Father laid up in store for his children the materials from which coal has been formed; He knew what would be their wants, and provided for them. Without this provision how deplorable would be our condition! No tree would gladden us with its leafy verdure or refresh us by its shade. The mountains would not be crowned with waving forests, or the hills with beauty. Our necessities would have required their sacrifice, and the earth would be desolate. Amalgam is a special providence. It has come in the order of Divine beneficence to supply an imperative need. Mastication is essential to the health and continuance of our race, and without Amalgam myriads of human teeth, that are now accomplishing their appropriate work, would have utterly perished. If in the warmth and light that emanate from the black product of the mine, we recognize the providing hand, why shall we not also behold it in the means that enable us to perform the functions essential to nourishment and life? Amalgam, like the Rubber Dam is one of the best aids to the highest professional achievement. Like the dental file, it has no faults in itself, and wherever skillfully used works out its own praise, and its capacity for usefulness is truly marvelous.

